# p-Coumaric acid regulates macrophage polarization in myocardial ischemia/reperfusion by promoting the expression of indoleamine 2, 3-dioxygenase

**DOI:** 10.1080/21655979.2021.2001924

**Published:** 2021-11-29

**Authors:** Na Li, Xueyuan Guo, Rui Li, Jian Zhou, Fangfang Yu, Xianliang Yan

**Affiliations:** a Department of Cardiology, Beijing Key Laboratory of Hypertension, Beijing Chaoyang Hospital, Capital Medical University, China; b Department of Cardiology, Beijing Anzhen Hospital, Capital Medical University, Beijing Institute of Heart Lung and Blood Vessel Diseases; c Department of Health Care, China-Japan Friendship Hospital, Beijing, China; d Department of Radiology, Beijing Chaoyang Hospital, Capital Medical University

**Keywords:** P-coumaric acid, macrophage polarization, myocardial ischemia/reperfusion, IDO

## Abstract

Macrophage infiltration is a hallmark pathological change observed in early stage myocardial ischemia/reperfusion (MI/R) injury and one of the main causes of myocardial damage. Here, we investigated the effects of p-Coumaric acid (p-CA) on macrophage polarization following MI/R injury and its mechanisms. *In vitro*, p-CA decreases the expression of LPS/IFN-γ-induced M1 macrophage markers (TNF-α, IL-6, iNOS and CCL2) and increases IL-4-induced M2 macrophage markers (IL-10, CD206, Arg1 and Mrc) in mouse bone marrow-derived macrophages (BMDMs). Additionally, p-CA elevated indoleamine 2, 3-dioxygenase (IDO) protein expression levels, M2 macrophage polarization and M2 macrophage markers through IL-4. In contrast, repression of IDO attenuated p-CA functions regulating BMDMs through IL-4. *In vivo*, IDO expression was downregulated in mouse hearts subjected to MI/R injury. Treatment of p-CA increased IDO expression in the hearts of MI/R mice. Functionally, p-CA decreases M1 macrophage markers, the number of M1 macrophages and inflammation around heart tissue following MI/R injury. Importantly, p-CA reduces cardiomyocyte apoptosis caused by MI/R. Altogether, our study identified that p-CA modulates macrophage polarization by promoting IDO expression and that p-CA attenuates macrophage-mediated inflammation following MI/R by promoting M2 macrophage polarization through IDO.

## 1. Introduction

Acute myocardial infarction (AMI) is one of the main causes of death. However, with the use of percutaneous coronary intervention (PCI), the mortality rate of AMI patients is decreasing yearly [1,2]. However, coronary artery reflow reconstructed by PCI treatment also leads to new injuries, including myocardial ischemia reperfusion (MI/R) injuries (MI/R) [3]. Immune activation plays an important role in MI/R injury. When the blood flow of the ischemic myocardium is restored, damaged cardiomyocytes release endogenous danger signals to recruit inflammatory cells, initiate/amplify the local inflammatory response and cause secondary damage to cardiomyocytes [4,5].

Monocytes and macrophages play an important role in the inflammatory response induced by MI/R [4,5]. In the perfused heart tissue, macrophages are mainly classified as M1 and release a variety of pro-inflammatory cytokines and chemokines that induce inflammation and promote the phagocytosis of dead cells [6,7]. With prolonged reperfusion time, M1 macrophages aggravate tissue damage and myocardial apoptosis [8]. In addition, the macrophages gradually become polarized toward M2, and M2 macrophages synthesize and release anti-inflammatory cytokines, immunosuppressive factors and a variety of growth factors that inhibit inflammation and promote myocardial tissue repair [9,10]. Therefore, it is important to suppress MI/R injury caused by excessive inflammation through promoting the conversion of macrophages from M1 to M2.

*p*-Coumaric acid (p-CA) is a derivative of cinnamic acid found in free form in fruits, vegetables and traditional Chinese medicines. It has many pharmacological functions, such as scavenging free radicals [11], inhibiting lipid peroxidation [12,13], anti-inflammatory responses [14,15], immune regulation [16] and plays a key role in neuroprotection [17]. Based on previous studies, we assumed that p-CA attenuates MI/R injury since p-CA reduces doxorubicin-induced oxidative stress [18], inhibits brain injury caused by ischemia-reperfusion [17] and regulates indoleamine 2,3-dioxygenase (IDO) expression in mouse immune cells [19]. Overexpression of IDO promotes polarization of dTHP-1 cells into M2 macrophages [20]. Therefore, p-CA regulates the polarization of macrophages by regulating the IDO expression, thereby reducing MI/R damage caused by macrophage-mediated inflammation. In this study, we confirmed this hypothesis to show that p-CA promotes the polarization of M2 macrophages in MI/R mouse heart tissues by promoting IDO expression.

## 2. Materials and methods

### 2.1 MI/R mouse model

All animal studies were performed according to the National Institutes of Health Guidelines for the Use of Laboratory Animals and were approved by the Ethics Committee of Beijing Chaoyang Hospital, Capital Medical University. C57BL/6 J male (n = 70, 8–10 weeks old) mice were maintained in pathogen-free facilities (24 ± 2°C and 45 ± 15% humidity with a 12/12 h light-dark cycle). After a week of acclimatization, mice were utilized for developing the MI/R model as previously described [21,22]. In brief, the mice were anesthetized by intraperitoneal injection of sodium pentobarbital (45 mg/kg), and the heart was exposed through an incision in the left chest. Next, a 6.0 silk suture slipknot ligated around the left anterior descending (LAD) of the coronary artery was generated to induce myocardial infarction for 30 minutes. Mice were euthanized after perfusion for 12, 24 or 48 hours. Mice in the sham group experienced surgery that exposed the heart, but LAD ligation was not performed. Two weeks before establishing MI/R model, mice were treated orally with 100 mg/kg of p-CA (C9008, Aladdin, USA) every day until they were euthanized.

### 2.2 Preparation of BMDMs

Bone marrow-derived monocytes were isolated from C57BL/6 J male mice (10–12 weeks of age) and were differentiated into BMDMs as previously described [23]. Briefly, mice were sacrificed by cervical dislocation, and bone marrow-derived cells were harvested from the femur or tibia. Bone marrow-derived cells were cultured in DMEM medium supplemented with 10% FBS and 1% penicillin/streptomycin for 12 hours before non-adherent cells were collected. Bone marrow-derived monocytes were differentiated into BMDMs using 40 ng/mL of GM-CSF (SRP3201, MERCK, USA) for 8 days. Lastly, BMDMs were polarized into M1 macrophages using 100 ng/mL of LPS and 20 ng/mL of IFN-γ for 24 hours. BMDMs were polarized into M2 macrophages using 20 ng/mL of IL-4 for 24 hours.

### 2.3 RNA extraction and qPCR

Total RNA was extracted from BMDMs and heart tissues using RNAiso plus (15,596,026, ThermoFisher Scientific, USA). Next, cDNA was performed using the First Strand cDNA Synthesis Kit (11,801–025, OriGene, USA) according to instructions provided by the manufacturer [24]. Quantitative reverse transcription polymerase chain reactions (qPCRs) were performed as described by the manufacturer (A6001, Promega, USA) using the applied biosystems PCR system. PCR primers are listed in Table 1.

**Table 1.** Sequence of qPCR primers

Gene	Sequence (5ʹ-3ʹ)
TNF-α	Forward: CCTGTAGCCCACGTCGTAG
	Reverse: GGGAGTAGACAAGGTACAACCC
IL-6	Forward: CTCCCAACAGACCTGTCTATAC
	Reverse: CCATTGCACAACTCTTTTCTCA
IL-10	Forward: CTGCCTTCAGCCAGGTGAAG
	Reverse: GGCCATGCTTCTCTGCCTGG
Mrc	Forward: CACCAAGCTGAACTTGAGCG
	Reverse: CGTGGCTTTGGGCTCCTC
iNOS	Forward: GTTCTCAGCCCAACAATACAAGA
	Reverse: GTGGACGGGTCGATGTCAC
CCL2	Forward: GGCATCCCTCTACCCAAGAC
	Reverse: GGGCGTTAACTGCATCTGGA
Arg1	Forward: CTCCAAGCCAAAGTCCTTAGAG
	Reverse: GGAGCTGTCATTAGGGACATCA
CD206	Forward: GCTTCCGTCACCCTGTATGC
	Reverse: TCATCCGTGGTTCCATAGACC
β-actin	Forward: AGCCCATCCTTCGAGTACAAA
	Reverse: TCTTGGTGCGATAACTGGTGG

### 2.4 Flow cytometry

Red blood cells were removed from a cardiac tissue single-cell suspension, counted and then incubated for 30 minutes on ice with one of the following antibodies: CD45-Pacific Blue (50–113-811, Invitrogen, USA), CD11b-APC-Cy7 (MABF512MI, MilliporeSigma, USA), Gr-1-APC (RM3005, Invitrogen, USA), CD86-PE-cy7 (11,527, Cell Signaling Technology, USA) or CD206-PE (12–2069-41, eBioscience, USA). Lastly, flow cytometry was used to detect the fluorescence and Flowjo 7.6.2 was used to analyze data.

### 2.5 Immunoblotting

As previous study, RIPA lysis buffer was used to extract total protein from mouse BMDMs and heart tissues [25]. Protein concentration was detected using a BCA kit. Cell or tissue lysates were separated using 10% SDS-PAGE. After the protein transfer step, PVDF membranes (LC2002, ThermoFisher) were blocked with 5% skimmed milk and then membranes were probed with primary antibodies against IDO (86,630, CST, USA), Bax (ab32503, Abcam, UK) or Bcl2 (ab196495, Abcam, UK). β-Actin served as a loading as control. After three washes in PBS buffer, secondary antibodies were incubated for 1 hour at room temperature. Lastly, proteins were visualized using ECL solution (WBKLS0100, Beijing Xinjingke Biotechnologies Co., Ltd., China). Blots were analyzed using Image J 3.0 (IBM, USA).

### 2.6 Repression of IDO

Small interfering RNAs (siRNAs) were used to repress IDO expression. A total of 50 nmol/l of siRNAs (5ʹ-UAGUCAGAAGCAUAGUGAGCA-3ʹ) were transfected into 2.5 × 106 BMDMs using Lipofectamine 2000 based on manufacturer protocols. The experiments were performed 72 hours after transfection.

### 2.7 Immunochemistry

Heart tissues were harvested 24 hours after reperfusion and 5 μM histological sections were prepared. Next, a IDO (86,630, CST, USA) primary antibody was added to tissues before washing three times with PBS containing tween 20. Then, a secondary antibody (Goat Anti-Rabbit IgG H&L (HRP) (ab6721, abcam, UK)) was incubated with tissue samples for 2 hours at room temperature. Lastly, the number of positive cells staining with IDO were counted using a Leica TCS SP5 microscope (Leica microsystem) containing LAS AF Lite 4.0 image browser software.

### 2.8 Inflammatory cytokines and caspase 3 activity assays

Heart tissues were harvested 24 hours after reperfusion and homogenate was prepared on ice using a glass homogenizer. Caspase 3 activity was detected using a Caspase 3 Activity Assay Kit (C1116, Beyotime, China) based on manufacturer instructions. To detect TNF-α, IFN-γ, IL-4 and IL-10 levels, the Mouse TNF alpha ELISA kit (ab208348, abcam, UK), IFN-γ ELISA Kit (ab100689, abcam, UK), IL-4 ELISA Kit (ab100710, abcam, UK) and IL-10 ELISA Kit (ab255729, abcam, UK), were used, respectively.

### 2.9 TUNEL staining to detect apoptosis of cardiomyocytes

Heart tissues were harvested 24 hours after reperfusion and 5 μM histological sections were prepared using the One Step TUNEL Apoptosis Assay Kit (C1088, Beyotime, China) based on manufacturer instructions. Briefly, sections were washed twice in PBS and 0.5% Triton X-100 was used to permeate cells after the section was fixed for 40 minutes using 4% paraformaldehyde. Lastly, 50 μl of TUNEL detection solution was added to the sections and incubated at 37°C for 60 minutes in the dark. The number of TUNEL positive cells was counted using a Leica TCS SP5 microscope (Leica microsystem) containing LAS AF Lite 4.0 image browser software.

### 2.10 Statistical analyses

SPSS 20.0 (IBM, USA) software was used to analyze data. Data were depicted as mean ± SD, and one-way ANOVA with a Turkey test as a post hoc test was used to compare the differences between multiple groups. P < 0.05 indicated statistical significance

## 3 Results

Herein, we study the effect of p-CA on the polarization of macrophages, and we hypothesized that p-CA reduces MI/R damage by promoting the activation of M2 macrophages. In the present study, we first study the effect of p-CA on the polarization of BMDM in vitro and found that p-CA promotes M2 macrophages polarization by increasing IDO expression. Next, we checked the effect of p-CA on the expression of IDO protein and macrophages polarization in MI/R mouse model and found that p-CA attenuated macrophage-mediated inflammation following by MI/R through promoting M2 macrophage polarization via increasing IDO expression.

## 3.1 p-CA regulates macrophage polarization in mouse BMDMs

**Figure 1.**
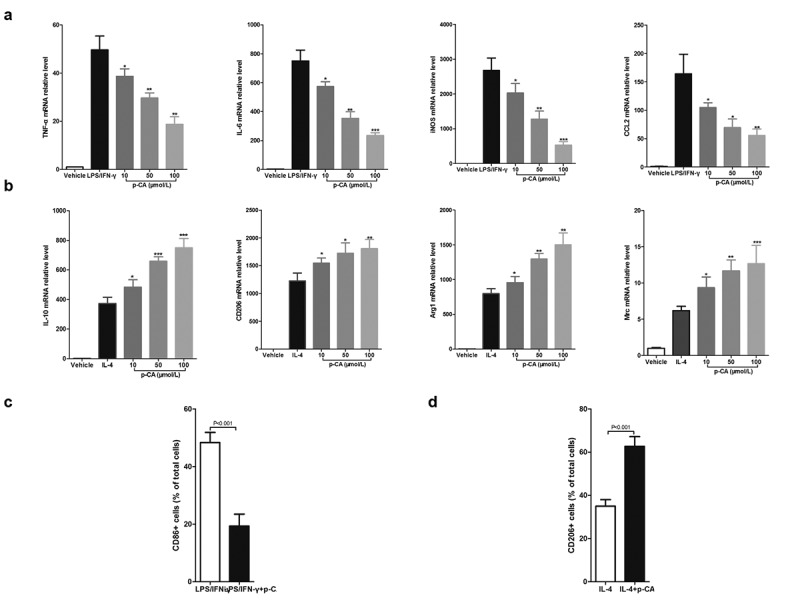
Effect of p-CA on the polarization of mouse BMDM in vitro. A, 24 hours after being stimulated with p-CA (10, 50 and 100 μmol/L), LPS (100 ng/mL) and IFN-γ (20 ng/mL), qPCR analysis was used to detected the expression of M1 macrophages markers (TNF-α, IL-6, iNOS and CCL2) in BMDM; B, 24 hours after being stimulated with p-CA (10, 50 and 100 μmol/L) or IL-4 (20 ng/mL), qPCR analysis was used to detected the expression of M2 macrophages markers (IL-10, CD206, Arg1 and Mrc) in BMDM; C-D, 24 hours after being treated with different methods, BMDM were harvested to analyze M1 (CD86) (c) and M2 (CD206) (d) macrophages markers using flow cytometer. Data was expressed as (mean ± SD) from 3 independent tests, and P value was calculated by post-hoc comparisons in (a) and (b), and by student’s t test in (c) and (d). * was P < 0.05, ** was P < 0.01 and *** was P < 0.001 vs LPS+IFN-γ group

Mouse BMDMs have been used as the model for studying macrophage polarization. When treated with LPS (100 ng/mL) and IFN-γ (20 ng/mL) for 24 hours, BMDMs will polarize into M1 macrophages. BMDMs will polarize into M1 macrophages after being treated with IL-4 (20 ng/mL) for 24 hours. To investigate the effects of p-CA on macrophage polarization, we first treated BMDMs with LPS (100 ng/mL), IFN-γ (20 ng/mL) and different concentrations of p-CA (10, 50 and 100 μmol/L) for 24 hours. qPCR analysis indicated that p-CA significantly decreased the expression of LPS/IFN-γ-induced M1 macrophage markers (TNF-α, IL-6, iNOS and CCL2) in BMDMs (Figure 1a). Similarly, BMDMs were treated with IL-4 (20 ng/mL) and different concentrations of p-CA (10, 50 and 100 μmol/L) for 24 hours to find that p-CA significantly increased IL-4-induced M2 macrophage markers (IL-10, CD206, Arg1 and Mrc) in BMDM (Figure 1b). In addition, BMDMs were harvested to analyze M1 (CD86) and M2 (CD206) macrophage markers using flow cytometer, 24 hours after treatment. We found that p-CA significantly decreased CD86 positive cells (Figure 1c) and increased CD206 positive cells (Figure 1d).

## 3.2 p-CA promotes M2 macrophage polarization by increasing IDO expression

**Figure 2.**
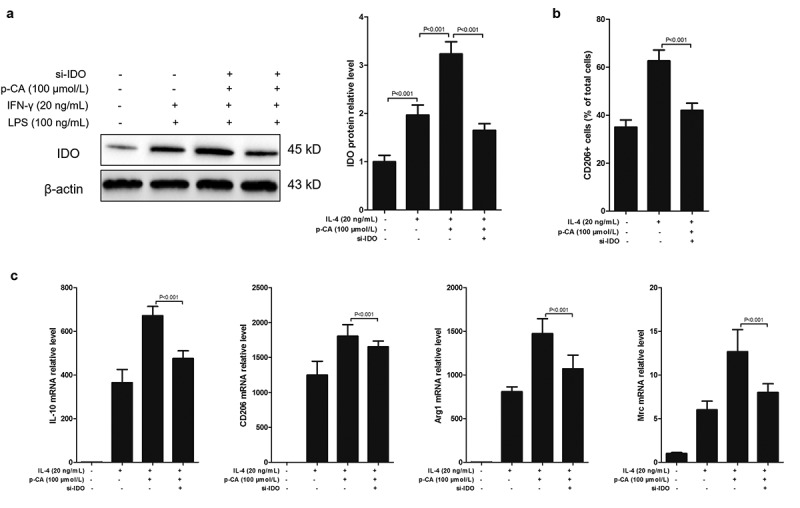
Inhibition of p-CA on LPS-induced M1 macrophage polarization by inhibiting IDO expression. A-C, after 24 hours of stimulation in different ways, BMDM were harvested to analyze IDO protein expression using Western blot, to analyze CD206 positive macrophage using flow cytometer, and to detect the expression of M2 macrophages markers (IL-10, CD206, Arg1 and Mrc) using qPCR. Data was expressed as (mean ± SD) from 3 independent tests, and P value was calculated by post-hoc comparisons in (a) and (b), and by student’s t test in (c)

p-CA inhibits the expression of IDO in mouse immune cells [19], and IDO plays an important role in the polarization of THP-1 (a cell line widely used as a model for monocytes/macrophages differentiation) [20]. Therefore, we assumed that p-CA induces M2 macrophage polarization by promoting IDO expression. To test this hypothesis, we first tested the effects of p-CA on the expression of IDO induced by IL-4 to find that p-CA treatment significantly increased IDO protein expression levels through IL-4 in BMDMs. Knockdown of IDO using siRNAs significantly reversed the effects of p-CA promoting IDO expression (Figure 2a). Importantly, knockdown of IDO significantly reversed the M2 macrophage ratio induced by p-CA (Figure 2b). At the same time, we also detected M2 macrophage markers, such as IL-10, CD206, Arg1 and Mrc, and qPCR analysis show that knockdown of IDO also significantly decreased the expression of IL-10, CD206, Arg1 and Mrc mRNAs promoted by p-CA (Figure 2c). Altogether, these results suggested that p-CA promotes IL-4-induced M2 macrophage polarization by increasing IDO expression.

## 3.3 p-CA increases IDO expression caused by MI/R injury

**Figure 3.**
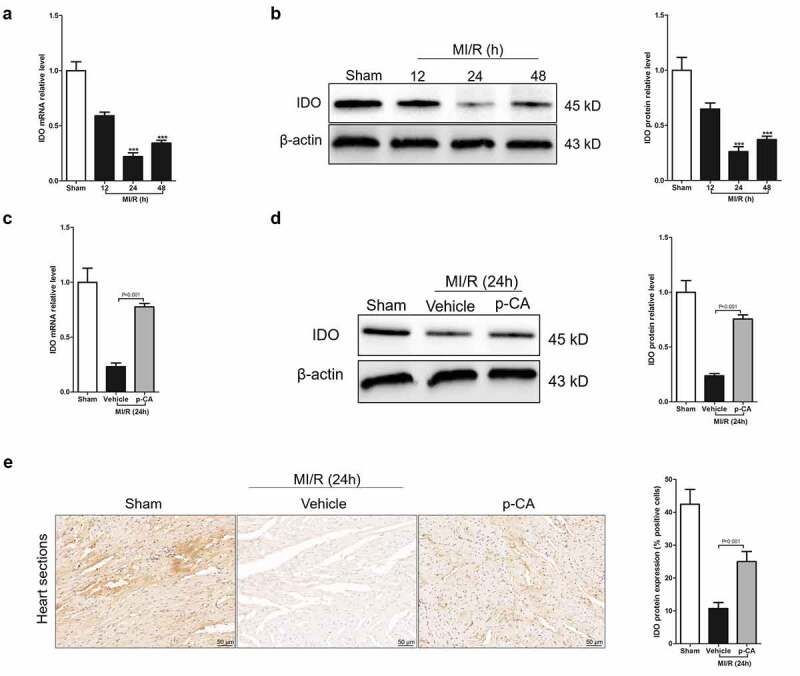
Effect of p-CA on elevated IDO expression in the heart tissues caused by MI/R. A-B, we harvested the mouse heart tissues following 12 h, 24 h and 48 h of MI/R injury, and then analyze IDO gene expression using qPCR (a) and immunoblotting (b); C-E, mice were treated with100 mg/kg of p-CA prior to MI/R injury, and mouse heart tissues were harvest following 24 h of MI/R injury to be analyzed IDO gene expression using qPCR (c), immunoblotting (d) and immunochemistry (e) for studying the effect of p-CA on IDO expression in MI/R mice. Data was expressed as (mean ± SD) from 7 mice per group, and P value was calculated by post-hoc comparisons in (A) and (B), and by student’s t test in (C), (D) and (E). *** was P < 0.001 vs Sham group

Macrophage-mediated inflammation is a key cause of myocardial damage resulting from MI/R [5]. The increased polarization of M1 macrophages causes macrophage-mediated inflammation [26]. p-CA regulates macrophage polarization by increasing IDO expression. Theoretically, if IDO expression decreases in MI/R mice, p-CA suppresses macrophage-mediated inflammation in MI/R mice by promoting IDO. Fortunately, we first detected dynamic changes of IDO mRNAs using RT-qPCR in mouse heart tissues at 12, 24 and 48 hours after reperfusion to find that IDO mRNA levels were rapidly down regulated and reached a plateau 24 hours after reperfusion (Figure 3a). Next, IDO mRNAs rapidly increased 24 hours after reperfusion and remained at a low level 48 hours after reperfusion. Similarly, dynamic changes in IDO protein levels in the heart tissues after reperfusion showed a rapid decrease in IDO a 24 hours after reperfusion, and then an increase in IDO protein (Figure 3b). To confirm that p-CA increased IDO expression induced by external factors *in vivo*, we compared the expression levels of IDO protein in MI/R heart tissues in mice with or without p-CA pre-treatment using qPCR, immunoblotting and immunohistochemistry. We found that p-CA pre-treatment significantly increased IDO protein expression in MI/R mouse hearts (Figure 3c-e).

## 3.4 p-CA regulates macrophage polarization in heart tissues of MI/R mice

**Figure 4.**
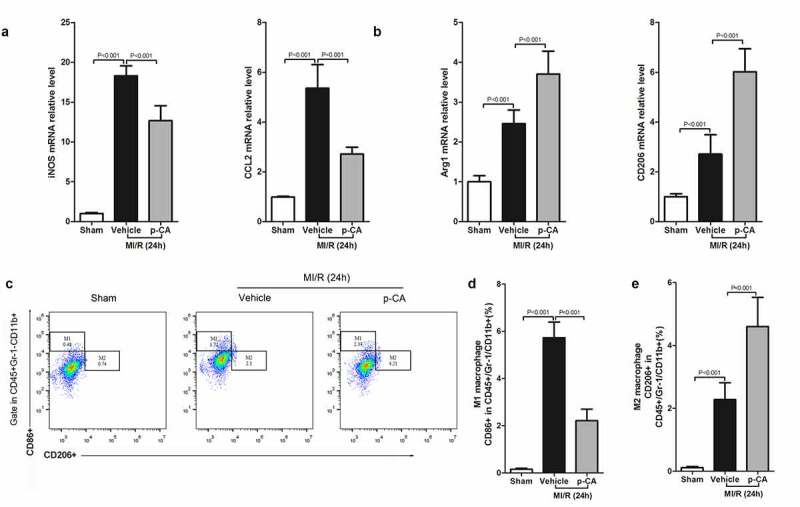
Effect of p-CA on macrophage polarization in the heart tissues caused by MI/R. A-B, 24 hours after MI/R injury, mice heart tissues were harvested to analysis M1 macrophages markers (iNOS and CCL2) (a) and M2 macrophages markers (CD206 and Arg1) (b) by qPCR; C-D, as described in (a and b), we prepared a single cell suspension of heart tissue, and used flow cytometer (c) to count the ratio of M1 macrophages (d) and M2 macrophages (e). Data was expressed as (mean ± SD) from 7 mice per group, and P value was calculated by student’s t test

**Figure 5.**
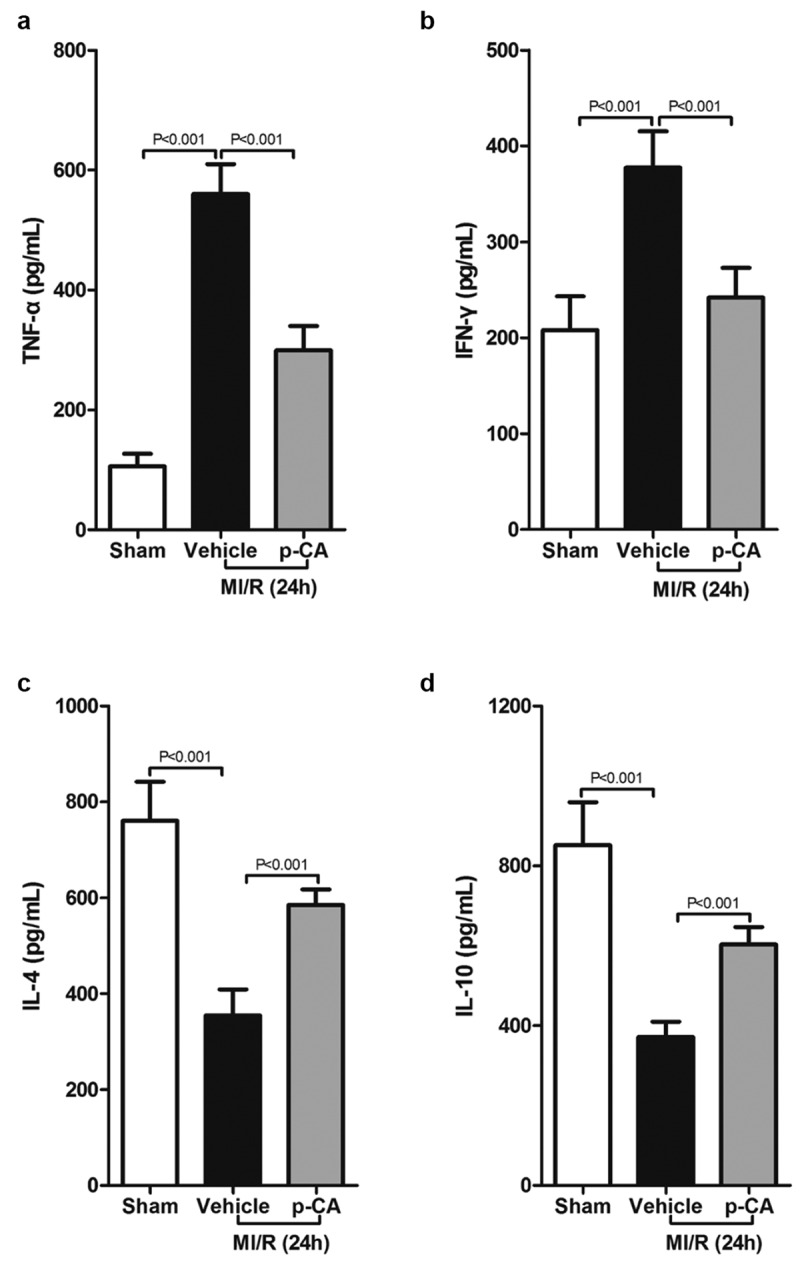
Effect of p-CA on inflammatory cytokines expression in the heart tissues caused by MI/R. A-D, 24 hours after MI/R injury, mice heart tissues were harvested to analysis TNF-α (a), IFN-γ (b), IL-4 (c) and IL-10 (d) by ELISA assay kit. Data was expressed as (mean ± SD) from 7 mice per group, and P value was calculated by student’s t test

We next investigated the effects of p-CA on macrophage polarization in the heart tissues of MI/R mice since p-CA significantly decreased the expression of IDO in MI/R mouse hearts. First, we determined the expression of M1 macrophage markers (iNOS and CCL2) and M2 macrophage markers (Arg1 and CD206), and found that both M1 and M2 markers were increased in MI/R mouse compared to the sham group. We also found that p-CA pre-treatment significantly decreased the expression of M1 macrophage markers (Figure 4a) and significantly increased the expression of M2 macrophage markers (Figure 4b). To further analyze the subtypes of macrophages infiltrating the heart tissues of MI/R mice, we prepared a single-cell suspension of heart tissues and detected the ratio of M1 macrophages (CD16+ CD45+ Gr-1-CD11b+) and M2 macrophages (CD206+ CD45+ Gr-1-CD11b+) using a flow cytometer (Figure 4c). Flow-cytometry analysis revealed that p-CA pre-treatment significantly decreased the ratio between M1 macrophages caused by MI/R injury (Figure 4d), while p-CA significantly increased the ratio of M1 macrophages caused by MI/R injury (Figure 4e). Moreover, we also found that p-CA pre-treatment significantly decreased TNF-α (Figure 5a) and IFN-γ (Figure 5b) mRNA expression levels and significantly increased IL-4 (Figure 5c) and IL-10 (Figure 5d) mRNA expression levels caused by MI/R injuries.

## 3.5 p-CA protects cardiomyocytes against MI/R injury

**Figure 6.**
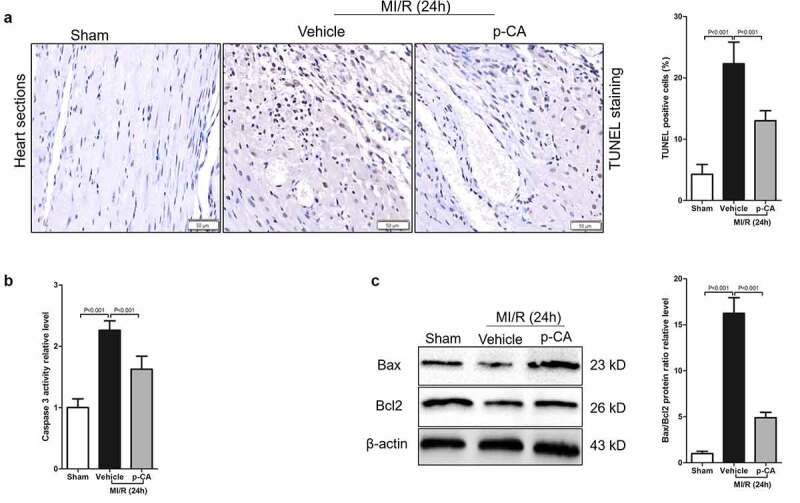
Effect of p-CA on cardiomyocyte apoptosis in the heart tissues caused by MI/R. A-C, 24 hours after MI/R injury, mice heart tissues were harvested to analysis cardiomyocyte apoptosis by TUNEL staining (a), to detect the caspase 3 activity by a kit (b), and to measure the expression of apoptosis-related proteins (Bax and Bcl2) by immunoblotting (c). Data was expressed as (mean ± SD) from 7 mice per group, and P value was calculated by student’s t test

Cardiomyocyte apoptosis caused by MI/R injury results in cardiac dysfunction and myocardial damage [27]. Here, we investigated the effects of p-CA on cardiomyocyte apoptosis caused by MI/R to find that the apoptosis of cardiomyocytes significantly increased and p-CA treatment significantly decreased cardiomyocyte apoptosis caused by MI/R, as shown by TUNEL staining (Figure 6a and b). p-CA not only significantly decreased the activity of caspase 3 caused by MI/R in mouse heart tissues (Figure 6b), but it also significantly decreased the increased ratio of Bax/Bcl2 protein expression caused by MI/R (Figure 6c). Overall, p-CA treatment decreased MI/R-induced cardiomyocyte apoptosis mouse heart tissues.

## 4. Discussion

In this study, we investigated the effects of p-CA on MI/R injury caused by macrophage-mediated inflammation, and we also touched on the mechanism behind this. Luckily, we found that p-CA suppressed M1 macrophage polarization by increasing the expression of IDO both *in vitro* and *in vivo*. This results in the attenuation of cardiomyocyte apoptosis caused by macrophage-mediated inflammation. Altogether, the results of this study indicated that p-CA is a potential drug that can prevent and treat MI/R injuries due to its ability to inhibit macrophage-mediated inflammation by repressing IDO expression.

p-CA is derived from natural plants, and its pharmacological activities have been reported as beneficial for MI/R. This compound contains both antioxidant and anti-inflammatory characteristics and has the ability to reduce atherosclerosis and inhibit apoptosis. p-CA contains a phenolic hydroxyl structure, providing hydrogen atoms to scavenge reactive oxygen species (ROS), terminating ROS free chain reactions and transferring electrons to form stable products through the conjugation of a benzene ring and double bond side chains [28,29]. Using different evaluation systems, coumaric acid has been shown to have free radical scavenging abilities, but its antioxidant activity is weaker than ortho-diphenol derivatives or ortho-position methoxy base derivatives, since there is only one phenolic hydroxyl group provided by p-CA [30,31]. For example, the antioxidant activity of p-CA is almost the same as butyl hydroxyanisole (BHA) and di-tert-butyl p-cresol (BHT), but better than vitamin C (VC) and vitamin E (VE) in the ABTS method. The scavenging ability of p-CA on O2 is lower than BHT, and the antioxidant activity of p-CA is second to VE based on an iron ion reduction ability experiment [30,31]. So far, we have not observed p-CA protected myocardial ischemia-reperfusion injury through anti-oxidative stress. There is no direct proof to show that the antioxidant activity of p-CA is responsible for the cardioprotective action observed in our study.

In addition, the anti-inflammatory effects of p-CA are closely related to its antioxidant activity. During inflammation, macrophages and neutrophils release ROS and cause tissue damage, while p-CA eliminates O2 induced by chemotactic peptides in human neutrophils, restores lipid peroxidation levels and induces endogenous antioxidant gene expression levels. This restored the antioxidant status of the body to near normal levels [32]. Importantly, p-CA inhibits the expression of inflammatory factors in a dose-dependent manner [33,34], including cyclooxygenase-2 (COX-2), inducible nitric oxide synthase (iNOS), tumor necrosis factor-α (TNF- α), interleukin-1β (IL-1β) and interleukin-6 (IL-6). In this study, we found that p-CA suppressed the polarization of M1 macrophages and promoted the polarization of M2 macrophages by inhibiting IDO expression *in vitro* and in the heart tissues of MI/R mice, thereby reducing MI/R injury caused by MI/R.

M1 macrophages promote inflammation by secreting pro-inflammatory factors (IL-1β, IL-6, IL-12, TNF-α), chemokines (MCP-1) and iNOS. M2 macrophages exert anti-inflammatory effects by producing large amounts of anti-inflammatory factors such as IL-10 and inhibiting the secretion of pro-inflammatory factors IL-12, IL-1, TNF-α [35,36]. Macrophages, especially M1 macrophages, have increased infiltration in tissues, which is an important pathological manifestation of tissue inflammation and one main causes of tissue damage [22,37]. The differentiation and activation of macrophages depend on specific growth and differentiation factors, receptors, signaling pathways and transcription factors [38,39]. Local microenvironmental signals play an important impact in the polarization of macrophages. Different microenvironmental signals induce macrophage polarization into two different states, including the immune activation or immune suppression states [40].

Indoleamine 2,3-dioxygenase (IDO) is the rate-limiting enzyme that catalyzes the degradation of tryptophan. An increase in IDO accelerates the metabolism of local tryptophan, exhausting tryptophan and resulting in the accumulation of its metabolite kynurenine, thereby changing the microenvironmental signals around macrophages [41]. Wang *et al*. found that IFN-γ up-regulated the expression of IDO at both the mRNA and protein levels in dTHP-1 cells in a concentration-dependent manner. In addition, this study also found that overexpression of IDO promoted the polarization of M2 macrophages, while knockdown of IDO promoted polarization of M1 macrophages in dTHP-1 cells [20]. A previous study also found that p-CA regulated the expression of IDO protein in mouse immune cells [19].

## Conclusion

Taken together, p-CA attenuates macrophage-mediated inflammation following MI/R by promoting M2 macrophage polarization through increasing IDO expression.

## Data Availability

The data used to support the findings of this study are available from the corresponding author upon request.
